# Evasion of Antiviral Innate Immunity by Porcine Reproductive and Respiratory Syndrome Virus

**DOI:** 10.3389/fmicb.2021.693799

**Published:** 2021-06-23

**Authors:** Tong-Yun Wang, Ming-Xia Sun, Hong-Liang Zhang, Gang Wang, Guoqing Zhan, Zhi-Jun Tian, Xue-Hui Cai, Chenhe Su, Yan-Dong Tang

**Affiliations:** ^1^State Key Laboratory of Veterinary Biotechnology, Harbin Veterinary Research Institute of Chinese Academy of Agricultural Sciences, Harbin, China; ^2^Department of Immunology, School of Basic Medical Sciences, Fujian Medical University, Fuzhou, China; ^3^Department of Infectious Disease, Renmin Hospital, Hubei University of Medicine, Shiyan, China

**Keywords:** antiviral innate immunity, PRRSV, PRRs, interferon, JAK-STAT, immune evasion, ISGs

## Abstract

Innate immunity is the front line for antiviral immune responses and bridges adaptive immunity against viral infections. However, various viruses have evolved many strategies to evade host innate immunity. A typical virus is the porcine reproductive and respiratory syndrome virus (PRRSV), one of the most globally devastating viruses threatening the swine industry worldwide. PRRSV engages several strategies to evade the porcine innate immune responses. This review focus on the underlying mechanisms employed by PRRSV to evade pattern recognition receptors signaling pathways, type I interferon (IFN-α/β) receptor (IFNAR)-JAK-STAT signaling pathway, and interferon-stimulated genes. Deciphering the antiviral immune evasion mechanisms by PRRSV will enhance our understanding of PRRSV’s pathogenesis and help us to develop more effective methods to control and eliminate PRRSV.

## Introduction

Since its discovery in the late 1980s, porcine reproductive and respiratory syndrome (PRRS) has become one of the most serious swine diseases in the world. PRRS virus (PRRSV), which causes PRRS, is an enveloped RNA virus belonging to the *Arteriviridae* family (Meulenberg, 2000). PRRSV is divided into two distinct genotypes: Type 1, or European-like (prototype Lelystad), and Type 2, or North American-like (prototype VR-2332) (Nan et al., 2017). PRRSV Types 1 and 2 were reclassified as two species belonging to the genus *Porartevirus*, PRRSV-1, and PRRSV-2, respectively, according to the current taxonomy (Adams et al., 2016). PRRSV-1 was first reported in Europe (Wensvoort et al., 1991) and then became epidemic in continental Europe and is currently in China (Chen et al., 2011), the United States (Fang et al., 2007), Canada (Dewey et al., 2000), South Korea (Lee et al., 2010), Thailand (Thanawongnuwech et al., 2004), and other countries. PRRSV-2 was first reported in the United States and then spread in some countries in Asia and Europe (Madsen et al., 1998; An et al., 2007; Shi et al., 2010b). PRRSV-1 is divided into Western Europe subtype I, Russia subtype I, subtype II, and subtype III (Shi et al., 2010a). PRRSV-2 is divided into nine lineages, lineag1∼9 (Shi et al., 2010a). PRRSV poses a threat to the global pig industry.

PRRSV encodes RNA replicates (ORF1a and ORF1b), four membrane-associated glycoproteins (GP2, GP3, GP4, and GP5), two unglycosylated membrane proteins (E and M), and a nucleocapsid (N) (Meulenberg, 2000; Music and Gagnon, 2010). ORF1a and ORF1b encode polyproteins that are processed into smaller protein products named non-structural proteins (Nsps), which are involved in viral RNA synthesis (Kroese et al., 2008; Li et al., 2014; Tang et al., 2016; Wang T. Y. et al., 2019), inducing replication-associated membrane rearrangement (Snijder et al., 2001; Posthuma et al., 2008), and modulating host immune responses (Fang and Snijder, 2010; Yoo et al., 2010; Sun et al., 2012; Ke and Yoo, 2017; An et al., 2020). Briefly, Nsp1 is involved in modulating subgenomic mRNA synthesis (Kroese et al., 2008), and Nsp2 and Nsp3 play an important role in replication-associated membrane rearrangement (Snijder et al., 2001; Posthuma et al., 2008). Nsp4 is the main protease responsible for the processing of viral polyproteins (Fang and Snijder, 2010). Nsp9 is an RNA polymerase, and Nsp10 is a helicase; both are key enzymes for arterivirus RNA synthesis and are reported as two key components for the virulence of highly pathogenic PRRSV (Li et al., 2014). Nsp11 is an endoribonuclease; however, its natural substrate is not identified yet (Fang and Snijder, 2010).

PRRSV infection damages innate and adaptive immune response in porcine. Cells of monocyte-macrophage lineage and monocyte-derived dendritic cells are susceptible to PRRSV infection (Loving et al., 2007). PRRSV dramatically destroys porcine immune organs, such as the thymus and bone marrow, which are very important for adaptive immune response (Butler et al., 2019; Wang G. et al., 2019; Wang et al., 2020). Furthermore, it is well recognized that cytokines regulate and participate in innate and adaptive immune responses (Banyer et al., 2000; Belardelli and Ferrantini, 2002; Kabelitz and Medzhitov, 2007). However, PRRSV infection induces alterations of immunoregulatory cytokines, which cause a prolonged delay in the activation of CTL and neutralizing antibody production. Thus, PRRSV infection always causes severe host immune response disorders, such as prolonged viremia, transiently diminishing T-cell immunity, and delayed protective antibody response (Molitor et al., 1997; Labarque et al., 2000; Lopez and Osorio, 2004; Xiao et al., 2004; Wu et al., 2020).

Innate immunity is the first-line host defense that limits the viral spread and regulates the adaptive immune responses. Viral pathogen-associated molecular patterns (PAMPs) are first recognized by host-pathogen recognition receptors (PRRs) and then triggers the associated signaling pathways, such as the interferon (IFN) regulatory factor (IRF) family members and the nuclear factor kappa-light-chain-enhancer of activated B cells (NF-κB) (Zheng, 2018; Zhu and Zheng, 2020). These transcription factors cooperate to modulate the expression of type I interferons (IFN-I) and, subsequently, evoke the downstream expression of IFN-stimulated genes (ISGs). However, PRRSV has evolved numerous strategies to evade innate antiviral immunity.

### Evasion of PRR Signaling Pathways

**FIGURE 1:**
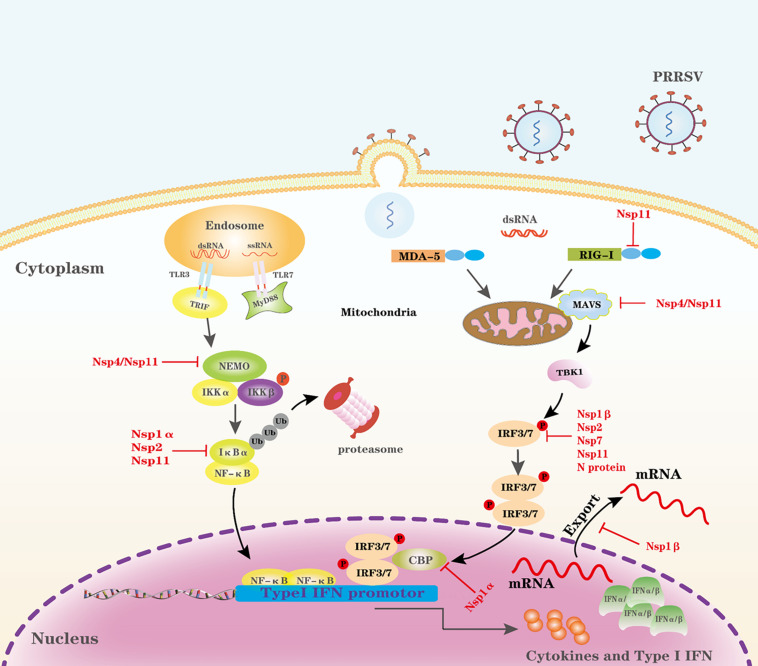
Evasion of the PRR-mediated IFN-I signal pathway by PRRSV. Cytosolic RNA sensors, such as TLR3, TLR7, RIG-1, and MDA-5, recognize PRRSV RNA in the cytosol and trigger IFN-I production by transmitting a series of signals. PRRSV proteins can target multiple steps in the RLR-mediated IFN-I signal pathway. CBP, CREB-binding protein; P, phosphate; Ub, ubiquitin.

The PAMPs of incoming viruses are first recognized by PRRs and activate an IFN response and proinflammatory cytokine responses during viral infection (Chow et al., 2015; Liu and Gack, 2020). Toll-like receptors (TLRs) and RIG-I-like receptors (RLRs) are two major PRRs in mammals that sense the RNA virus (Chow et al., 2015). Among all TLRs, TLR3 recognizes viral double-stranded RNA; TLR7 and TLR8 recognize viral single-stranded RNA (Alexopoulou et al., 2001; Heil et al., 2004). Porcine TLR3 and TLR7 are the best investigated TLRs in porcine cells. For PRRSV infection, expression of porcine TLR3, and TLR7 are upregulated and harbor the ability to elicit activation of IFN (Sang et al., 2008a,b; Liu et al., 2009; Miller et al., 2009). Activation of TLR3 by dsRNA results in the recruitment of Toll-interleukin 1 receptor domain-containing adapter inducing interferon-β (TRIF) through the adaptor protein myeloid differentiation factor 88 (MyD88) independent pathway (Fitzgerald et al., 2003). However, TLRs are limited in detecting viruses because they are expressed in a limited range of cell types (Chow et al., 2015). In contrast, the RLRs, RIG-I, and melanoma differentiation-associated protein 5 (MDA5) expressed in almost all cell types are cytoplasmic RNA helicases that recognize non-self RNA (Chow et al., 2015, 2018; Rehwinkel and Gack, 2020). Porcine RIG-I, MDA5, and mitochondrial antiviral signaling protein (MAVS, also known as IPS-1/VISA/Cardif) are important sensors/adaptors to produce type I IFN in the porcine innate immune system (Wang et al., 2008; Husser et al., 2011; Dong et al., 2013). RIG-I or MDA5 recognize intracellular dsRNA via DExD/H-box helicase domains. Caspase recruitment domains (CARDs) of RIG-I or MDA5 interact with the counterpart domains of MAVS (Kawai et al., 2005). Although different adaptors are utilized, both pathways converge to stimulate the two downstream kinases, Tank-binding kinase 1 (TBK1), and inhibitor of κB kinase ε (IKKε), resulting in the phosphorylation and activation of transcription factors, such as IFN regulatory factor 3 (IRF3), NF-κB, and AP-1 (Fitzgerald et al., 2003). These transcription factors coordinate in forming a transcriptionally competent enhanceosome that produces type I IFN and proinflammatory cytokine responses (Thanos and Maniatis, 1995). Type I IFNs are produced upon infection of animal cells with viruses, and they are responsible for the first line of defense during virus infection (De Maeyer and De Maeyer-Guignard, 1998; Bogdan, 2000). Type I IFNs have a broad and diverse impact on the priming of expansion and maturation of adaptive immunity (Bogdan, 2000; Theofilopoulos et al., 2005). PRRSV has evolved complex strategies to evade type I IFN restriction as illustrated in Figure 1, which is discussed in detail.

### Targeting RIG-I and MAVS

RIG-I and MDA5 are well-conserved cytoplasmic PRRs that detect viral RNAs and interact with the downstream adaptor MAVS and activate the antiviral signaling pathway (Wu and Hur, 2015). Both RIG-I and MAVS are critical for Type I IFN signaling. A previous study shows that PRRSV infection inhibits IFNβ production primarily by interfering with the MAVS activation in the RIG-I signaling pathway (Luo et al., 2008). Further studies demonstrate that Nsp4 and Nsp11 target RIG-I and/or MAVS. PRRSV Nsp4 is a 3C-like protease (3CLSP), cleaves MAVS at Glu268 and, thus, inhibits Type I IFN signaling (Dong et al., 2015). PRRSV Nsp11 decreases MAVS and RIG-I mRNA, and an endoribonuclease activity is critical for the antagonism (Sun et al., 2016).

### Targeting IFN Regulatory Factor 3 (IRF3)

IRF3 is a key node in the IFN signaling pathway, and it remains in the cytoplasm of unstimulated cells; however, when cells are infected by viruses, IRF3 is phosphorylated at various serine and threonine residues at the C terminus, and then phosphorylated IRF3 homodimerizes and translocates into the nucleus (Yoneyama et al., 2002; Hiscott et al., 2003). In addition, IRF3 C-terminal phosphorylation is required for association with the histone acetyltransferase nuclear proteins CBP [CREB (cyclic AMP responsive element binding)-binding protein] and p300 causing IRF3 to shuttle into the nucleus to induce transcription through distinct positive regulatory domains in the type I IFN promoters (Yoneyama et al., 1998; Hiscott et al., 1999). PRRSV antagonizes IRF3 by following strategies. First, PRRSV Nsp1β significantly blocks dsRNA-induced phosphorylation and nuclear translocation of IRF3 (Beura et al., 2010). Furthermore, PRRSV Nsp2 also antagonizes activation of IRF3 by inhibiting its phosphorylation and nuclear translocation, and the cysteine protease domain (PL2) of Nsp2 is required to antagonize IRF3 (Li et al., 2010). PRRSV Nsp11 is demonstrated to have an inhibitory effect on IRF3 activation, and this activity is endoribonuclease dependent (Shi et al., 2011). Last, PRRSV N protein similarly antagonizes IRF3 activation as Nsp1β and Nsp2, and it significantly abrogates dsRNA-induced IRF3 phosphorylation and nuclear translocation (Sagong and Lee, 2011). However, the underlying mechanisms to antagonize IRF3 activation by the above viral proteins are unclear because there is no report on whether these viral proteins interact directly or indirectly with IRF3.

### Targeting IFN Regulatory Factor 7 (IRF7)

Interferon regulatory factor 7 (IRF7) is a multifunctional transcription factor that was originally discovered during Epstein–Barr virus (EBV) infection, and it has been recognized as one of the major players in virally induced IFN signaling (Zhang and Pagano, 2002). Posttranslational modifications of IRF7 have important roles in regulating IRF7 activity, including phosphorylation and ubiquitination (Zhang and Pagano, 2002). PRRSV could downregulate the expression of IRF7 in pulmonary alveolar macrophages, and this activity is attributed to Nsp7 (Liu et al., 2019). However, the mechanism that PRRSV Nsp7 inhibits the expression of IRF7 is unclear, and this needs further investigation.

### CREB (Cyclic AMP Responsive Element Binding)-Binding Protein (CBP)

The CBP coactivator is a histone acetyltransferase; p300 and CBP are partners that cooperatively play a key role in transcriptional responses to disparate extracellular and intracellular signals (Giordano and Avantaggiati, 1999). CBP and p300 play multifunctional roles of the coactivator in transcriptional regulation, and in fact, many transcription factors have been reported to bind CBP (Goldman et al., 1997). PRRSV Nsp1α could degrade CBP in a proteasome-dependent manner, and its degradation prevents CBP recruitment for enhanceosome assembly, resulting in a blockage of IFN response (Kim et al., 2010). The Nsp1α contains three distinct functional motifs; a papain-like cysteine protease alpha (PCPα) motif, an N-terminal zinc finger motif (ZF1), and a recently discovered C-terminal zinc finger motif (ZF2). ZF1 is a required component of Nsp1α to suppress IFN production. Wild-type Nsp1α localizes in both the nucleus and the cytoplasm, but the ZF1 mutants lose their IFN suppression by inhibiting Nsp1α shuttling from the cytoplasm to the nucleus (Han et al., 2013; Han and Yoo, 2014). CBP degradation is most likely the primary mechanism for IFN suppression mediated by PRRSV (Han et al., 2013; Han and Yoo, 2014).

### NF-κB

#### Targeting NF-κB Essential Modulator (NEMO/IKKγ)

Nuclear factor-κB (NF-κB) essential modulator (NEMO), a component of the inhibitor of κB kinase (IKK) complex (which includes two kinases, IKKα and IKKβ), regulates NF-κB signaling by binding to ubiquitin chains (Kensche et al., 2012). IKK complex is a critical regulator for its downstream signaling by phosphorylating IκBα, leading to its subsequent degradation by the ubiquitin-proteasome system (Hayden and Ghosh, 2008). To activate the IKK complex, NEMO binding to ubiquitin chains is a critical step in linking upstream ubiquitin signals (Kensche et al., 2012). PRRSV nsp4 is an antagonist of NEMO, which cleave NEMO at multiple sites (Huang et al., 2014; Chen et al., 2019). Further study demonstrates that aspartic acid at residue 185 modulates Nsp4 catalytic activity, and this activity is responsible for NEMO cleavage (Wei et al., 2020). Linear ubiquitination targeting NEMO plays a critical role in the regulation of NF-κB signaling. PRRSV Nsp11 cooperates with swine linear ubiquitination-specific deubiquitinase, ovarian tumor domain deubiquitinase with linear linkage specificity (OTULIN) to remove linear ubiquitination of NEMO, which subsequently blocks the activation of NF-κB signaling (Su et al., 2018).

#### Targeting I-Kappa-B-Alpha (IκBα)

NF-κB plays a critical role in coordinating the expression of numerous genes that regulate immune responses (Li and Verma, 2002). NF-κB proteins are present in the cytoplasm in association with inhibitory proteins known as inhibitors of NF-κB (IκBα). After activation by upstream signals, the IκB proteins become phosphorylated, ubiquitylated, and subsequently degraded by the K48-linked ubiquitin-proteasome pathway (Li and Verma, 2002). The degradation of IκB, thus, relives NF-κB proteins to translocate to the nucleus and regulate the transcription of many genes, including the production of IFN-I and inflammatory chemokines.

IκBα is an inhibitor of NF-κB, blocking nuclear translocation and DNA binding. For NF-κB activation, IκBα is phosphorylated by upstream kinases and then is ubiquitinated, leading to proteosome-mediated degradation. NF-κB is then released into the nucleus, where it activates a slew of genes involved in immune and inflammatory responses (Jacobs and Harrison, 1998; Seth et al., 2005). Nsp1α has been reported to counteract IκBα by inhibiting its phosphorylation, which is a key step for NF-κB activation, and a further study indicates that Met180 and C-terminal 14 amino acids of the Nsp1α are crucial for inhibitory activities (Song et al., 2010). PRRSV nsp2 has a cysteine protease domain at its N terminus that belongs to the ovarian tumor (OTU) protease family. The PRRSV Nsp2 OTU domain has ubiquitin-deconjugating activity, and this domain potently inhibits NF-κB activation by interfering with the polyubiquitination process of IκBα, preventing IκBα degradation (Sun et al., 2010). Except for Nsp2, PRRSV Nsp11 encodes a unique and conserved endoribonuclease (nidovirus-specific endoribonuclease, NendoU) with DUB activity that specifically removes Lysine 48 (K48)-linked polyubiquitin chains of IκBα (Wang et al., 2015). Overall, PRRSV engages Nsp1α, Nsp2, and Nsp11 to interfere with the polyubiquitination process of IκBα.

#### Evasion of IFNAR-JAK-STAT Pathway

A typical PRRSV infection in pigs is characterized by delayed production and low titer of virus-neutralizing antibodies as well as weak cell-mediated immune response. One possible explanation for PRRSV-induced weak protective immune responses is that PRRSV may disrupt cytokine-mediated JAK-STAT signaling (Yang and Zhang, 2017). Various cytokines activate a JAK-STAT signaling pathway; nearly 40 cytokine receptors signal through combinations of four JAK (JAK1, JAK2, JAK 3, and Tyk2) and seven STAT (STAT1–STAT7) family members, which are involved in the regulation of cell growth, proliferation, differentiation, apoptosis, angiogenesis, immunity, and inflammatory response (Rawlings et al., 2004; Murray, 2007). Cytokines first bind their receptors, which cytoplasmic domains are associated with JAK tyrosine kinases, and then two JAKs are brought into proximity allowing trans-phosphorylation, which further induces the multimerization (homodimers or heteromultimers depend on the type of cytokine) of receptor subunits. The activated JAKs subsequently phosphorylate the major substrates, STATs, which are latent transcription factors. STATs contain a conserved tyrosine residue near the C-terminus that is responsible for phosphorylation by JAKs. STATs form dimers by interacting with a conserved SH2 domain. Phosphorylated STATs dimers enter the nucleus mediated by importin α-5 (also known as karyopherin-α1, KPNA1) and the Ran nuclear import pathway allows phosphorylated STATs dimers to enter the nucleus and bind specific regulatory sequences to modulate target gene expression (Imada and Leonard, 2000; Rawlings et al., 2004). Negative regulators of the JAK/STAT pathway fall into three categories: suppressors of cytokine signaling (SOCS), protein inhibitors of activated stats (PIAS), and protein tyrosine phosphatases (PTPs) (Starr and Hilton, 1999; Greenhalgh and Hilton, 2001).

PRRSV uses multiple strategies to antagonize the JAK-STAT signaling pathway (Yang and Zhang, 2017). PRRSV infection is shown to disrupt the activation and signaling pathway of type I IFNs by inhibiting ISGF3 nuclear translocation (Patel et al., 2010). PRRSV Nsp1β induces karyopherin-α1 (KPNA1) degradation, which is a critical factor responsible for ISGF3 nuclear translocation; therefore, Nsp1 prevents IFN-induced ISGF3 complex nuclear translocation (Chen et al., 2010; Patel et al., 2010). Further study illustrates that Nsp1β Valine-19 is essential for inducing degradation of KPNA1 (Wang et al., 2013b). N protein expression leads to inhibition of IFN-induced elevation of STAT2 levels and ISGF3 nuclear translocation. However, the detailed mechanism was not well established (Wang et al., 2013a). PRRSV infection-induced STAT2 degradation through the ubiquitin-proteasome degradation pathway and Nsp11 is shown to interact with STAT2 directly and be responsible for STAT2 degradation (Yang et al., 2019). Interestingly, Nsp11 mediated STAT2 degradation is not dependent on the endoribonuclease activity, and amino acid residue K59 in nsp11 is required for STAT2 degradation (Yang et al., 2019). A recent study also demonstrates that Nsp11 interacts with interferon regulatory factor 9 (IRF9), which impairs the formation and nuclear translocation of ISGF3, and this activity is also independent of endoribonuclease activity (Wang D. et al., 2019).

**FIGURE 2:**
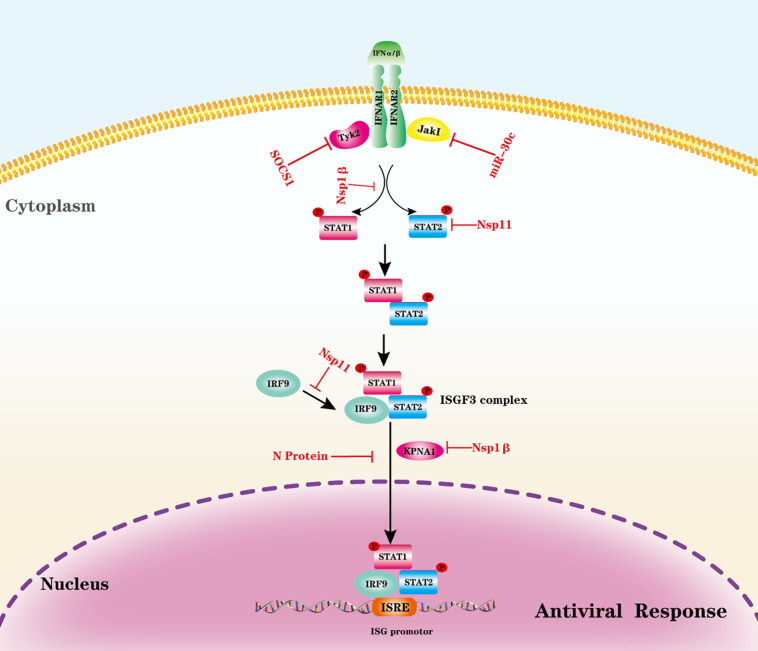
Evasion of the IFNAR-JAK-STAT signaling pathway by PRRSV. The antiviral activities of IFN-I and other cytokines are initiated by binding to their receptors to trigger a signaling cascade. Viral proteins from PRRSV interact with indicated adaptors to block signal transduction. P, phosphate.

Interestingly, PRRSV also modulates the JAK/STAT pathway by other strategies, such as microRNA and SOCS. PRRSV infection upregulates a host microRNA, miR-30 c, to target JAK1 and, subsequently, promotes PRRSV replication (Zhang et al., 2016). PRRSV also upregulates SOCS1, a negative regulator for the JAK/STAT pathway (Wysocki et al., 2012). The PRRSV N protein can increase SOCS1 activity, and nuclear localization signal-2 (NLS-2) is required for SOCS1 induction (Luo et al., 2020a). The evasion of the IFNAR-JAK-STAT signaling pathway by PRRSV is illustrated in Figure 2. With the continuous exploration in this field, we think more and more strategies by which PRRSV evades the JAK-STAT pathway will be uncovered.

#### Evasion of ISGs and Intrinsic Antiviral Proteins

**FIGURE 3:**
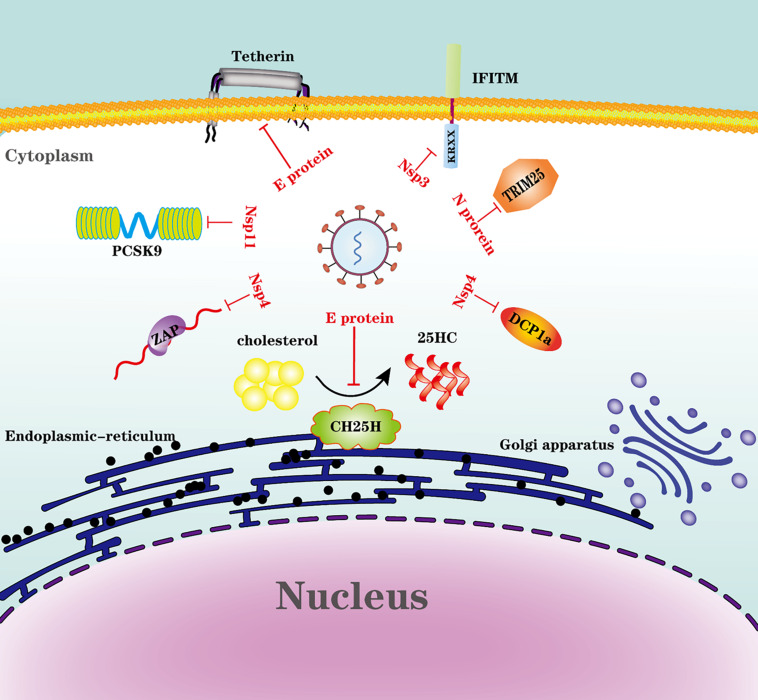
Evasion of ISGs and intrinsic antiviral proteins by PRRSV. Viral proteins from PRRSV engage multiple strategies to evade the restriction of ISGs or intrinsic antiviral proteins.

Many intrinsic antiviral proteins can inhibit PRRSV replication, but we only discuss several proteins antagonized by PRRSV. The evasion of ISGs and intrinsic antiviral proteins by PRRSV is illustrated in Figure 3.

#### Proprotein Convertase Subtilisin/Kexin Type 9 (PCSK9)

Proprotein convertase subtilisin/Kexin type 9 (PCSK9) is an enzyme that belongs to the subtilisin-like serine proteases family that participates in the proteolytic maturation of various proteins, such as hormones and cytokines (Seidah and Chretien, 1999). PCSK9 is a key component for plasma cholesterol metabolism, which controls low-density lipoprotein receptor (LDLR) levels by increasing LDLR degradation (Cohen et al., 2005; Maxwell et al., 2005). It is reported that PCSK9 can impede the replication of several viruses through different mechanisms. Dengue virus (DENV) infection induces the expression of PCSK9, which inhibits the recycling of LDL receptors and reduces uptake of LDL cholesterol in cells, compensatively; this cholesterol-deprived cell then activates and increases cholesterol synthesis in the endoplasmic reticulum, which subsequently decreases the expression of antiviral type I interferon genes (Gan et al., 2020). Hepatitis C virus (HCV) infection modulates the HCV receptors LDLR and CD81 on the liver cell surface, and PCSK9 modulates CD81 cell surface expression in an LDLR-independent manner (Labonte et al., 2009). For PRRSV, PCSK9 is induced by PRRSV infection in porcine alveolar macrophages at an early stage, and PCSK9 protein suppresses the replication of PRRSV by targeting the virus receptor CD163, which is important for PRRSV infection (Wang et al., 2018; Zhang et al., 2020). Nsp11 could antagonize PCSK9’s antiviral activity, and this activity is endoribonuclease activity-dependent (Zhang et al., 2020).

#### The Tripartite Motif-Containing 25 (TRIM25)

TRIM25 is an E3 ubiquitin ligase that is thought to be a crucial component in the activation of RIG-1 signaling. Recently, TRIM25 was identified as an RNA binding protein that may be essential for its function in innate immunity (Choudhury et al., 2020). The K63-linked ubiquitination of RIG-I by TRIM25 is needed to initiate the intracellular antiviral responses (Gack et al., 2007; Martin-Vicente et al., 2017). Influenza A virus NS1 interacts with TRIM25, thus blocking TRIM25 multimerization and RIG-I CARD domain ubiquitination (Gack et al., 2009). The severe acute respiratory syndrome coronavirus (SARS), and Middle East respiratory syndrome CoV (MERS-CoV) N protein inhibit TRIM25-mediated RIG-I ubiquitination, resulting in the inhibition of IFN production (Hu et al., 2017). PRRSV also uses N protein to interfere with TRIM25-RIG-I interaction by competitively interacting with TRIM25. N protein suppresses IFN-β production by inhibiting TRIM25 expression and TRIM25-mediated RIG-I ubiquitination (Zhao et al., 2019a). It seems like there is a common mechanism for nidoviruses to antagonize TRIM25. It is reported that a specific DENV lineage encodes sgRNA antagonizing TRIM25. DENV-2 produces subgenomic RNA during replication, which shows sequence-dependent binding to and prevention of TRIM25 deubiquitylation, which is a specific viral RNA-host protein interaction to suppress the innate immune responses (Manokaran et al., 2015); however, whether PRRSV has a similar mechanism to antagonize TRIM25 needs further exploration.

#### Zinc Finger Antiviral Protein, ZAP

Zinc-finger antiviral protein (ZAP), also known as zinc finger CCCH-type containing antiviral 1 (ZC3HAV1), is another ISG that was originally discovered as an antiretroviral factor (inhibition of retroviral RNA production by ZAP, a CCCH-type zinc finger protein) (Gao et al., 2002). ZAP in antiviral immunity is mainly based on its RNA binding property; in most known cases, ZAP recognizes viral RNA and recruits both the 5′ and 3′ mRNA RNA decay machinery to degrade the target RNAs, which is considered to be the principal antiviral mechanism mediated by ZAP (Zhu et al., 2011; Luo et al., 2020b; Wang and Zheng, 2020). Viral RNAs harbor the ZAP-responsive element (ZRE), a characteristic ZAP binding site; however, the corresponding ZRE position in each virus RNA sequence varies (Wang and Zheng, 2020). In PRRSV infection, ZAP is upregulated and then suppresses PRRSV replication at the early stage of replication; Nsp9 is reported to interact with ZAP (Zhao et al., 2019b). However, the mechanism that ZAP inhibits PRRSV replication is not well illustrated. Whether PRRSV harbors the ZRE or viral RNA degrades by ZAP is unclear. Interestingly, PRRSV could escape ZAP restriction by Nsp4, which cleaves ZAP dependent on its protease activity (Zhao et al., 2020). Further study reveals that serine 180 of Nsp4 is necessary for efficient degradation of ZAP, and the mutation at residue 180 is crucial for degradation of ZAP (Zhao et al., 2020).

#### IFITM1 and Tetherin

Interferon-inducible transmembrane proteins (IFITMs) are critical antiviral factors that belong to a family of small transmembrane proteins. IFITM proteins can impair broad-spectrum viral infection through multiple mechanisms, including limiting the viral entry, decreasing viral gene expression and viral protein synthesis, restricting viral assembly, and reducing viral infectivity (Liao et al., 2019). IFITM1 is one of the IFITMs. The potency and breadth of viruses restricted by IFITM1 are determined by its C-terminal non-canonical dibasic sorting signal KRXX, which suppresses certain viruses by regulating their intracellular dissemination (Li et al., 2015). PRRSV Nsp3 could counteract the restriction of IFITM1 by interaction with IFITM1 and then inducing the proteasome-dependent degradation of IFITM1 (Wang et al., 2014).

Tetherin is a type II single-pass transmembrane protein known as BST-2, CD317, or HM1.24. It has a cytoplasmic N-terminal region, followed by a transmembrane domain, a coiled-coil extracellular domain, and a glycosylphosphatidylinositol (GPI) anchor at the C-terminus (Kupzig et al., 2003). Tetherin inhibits viral replication by preventing newly formed virions from the host cell surface (Malim and Emerman, 2008; Neil and Bieniasz, 2009). PRRSV E protein could antagonize tetherin by interaction with tetherin and further partially remove tetherin away from the cell surface (Wang et al., 2014).

#### mRNA-Decapping Enzyme 1a (DCP1a)

The mRNA-decapping enzyme 1a (DCP1a) is a cofactor involved in the removal of the 5’-methylguanosine cap from eukaryotic mRNA in the granules known as processing bodies (P-bodies) (Sheth and Parker, 2003; Franks and Lykke-Andersen, 2008). DCP1a is involved in P-body formation, maintenance, and regulation (Franks and Lykke-Andersen, 2008). DCP1a also induces translational arrest by activating double-stranded RNA-dependent protein kinase (PKR) (Dougherty et al., 2014). Furthermore, DCP1a has recently been identified as an ISG (Schoggins et al., 2011, 2014). DCP1a can restrict poliovirus infection by inducing translational arrest (Dougherty et al., 2014). DCP1a has been demonstrated to inhibit PRRSV, whereas the mechanism is unclear (Tao et al., 2018). By counteracting DCP1a inhibition, PRRSV uses Nsp4 to cleave DCP1a, and the cleaved site is at glutamic acid 238 (E238) of porcine DCP1a; interestingly, this cleavage site is species-specific (Tao et al., 2018).

#### Cholesterol 25-Hydroxylase (CH25H)

Cholesterol-25-hydroxylase (CH25H) is one of the ISGs identified as a broadly antiviral ISG (Liu et al., 2013). CH25H is an endoplasmic-reticulum-associated enzyme catalyzing cholesterol oxidation to a soluble antiviral factor, 25-hydroxycholesterol (25HC) (Holmes et al., 2011). 25HC broadly inhibits enveloped viruses by blocking virus entry (Liu et al., 2013). For some viruses, including PRRSV, CH25H could inhibit viral replication through enzyme activity-dependent and independent mechanisms (Lv et al., 2019; Li et al., 2020). CH25H restricts PRRSV replication by inhibiting virus entry and degrading Nsp1α *via* the ubiquitin-proteasome pathway with K169 in the Nsp1 protein serving as the main ubiquitination location (Ke et al., 2017). PRRSV has also evolved several strategies to overcome the restriction of CH25H. First, PRRSV E protein interacts with CH25H and then degrades it via the ubiquitin-proteasome pathway, and the ubiquitination site is identified at Lys28 (Ke et al., 2019). Second, Nsp1β and Nsp11 mediate the degradation of CH25H via a lysosomal pathway with His159 in Nsp1β and NendoU involvement in Nsp11 playing critical roles in CH25H downregulation (Dong et al., 2018). However, CH25H has no interaction with Nsp1β or Nsp11, and the detail mechanisms by which Nsp1β and Nsp11 mediates CH25H degradation need further exploration (Dong et al., 2018).

## Future Perspective

Viruses have evolved multiple strategies to evade innate immune surveillance. This review summarizes how PRRSV engages several strategies to evade the porcine RNA sensing pathway of innate immune responses. However, the roles of DNA sensing pathways in PRRSV infection are unclear. Although there is no specific antagonism reported so far, the role of DNA sensors, such as IFI16, are reported to have an anti-PRRSV activity (Chang et al., 2019). There is emerging evidence showing the contribution of damaged host DNA, such as mitochondrial DNA, to innate immune responses against RNA viruses. Furthermore, PRRSV is highly genetically variable. How the genetic variations affect the immune modulation function of the viral proteins is the key to understanding the difference in virulence of different PRRSV strains, which needs further exploration in the future.

PRRSV has a potent spatiotemporal regulation ability to immune response. First, it evades TLR and RLR signaling to suppress the production of Type I IFNs, which are key components that modulate the development and maturation of adaptive immunity. Second, it also impairs antiviral response by targeting the IFNAR-JAK-STAT pathway. Last, PRRSV also evades ISGs and intrinsic antiviral proteins to promote viral replication. We think there are other undiscovered strategies for PRRSV to evade the innate immune response, and further investigation is needed.

In addition to the innate immune signal pathways mentioned above, unfolded protein responses, stress granules, and apoptosis are key components of cellular innate immunity. However, it seems that only UPR could be effectively counteracted by PRRSV (Gao et al., 2019). For stress granules and apoptosis, PRRSV infection induces but does not inhibit these responses (Catanzaro and Meng, 2019; Ruedas-Torres et al., 2020). Therefore, further studies focused on these directions will help us to comprehensively understand how PRRSV evades innate immune responses. Deciphering PRRSV evasion of innate immune responses will enhance our understanding of PRRSV’s pathogenesis and develop more effective methods to control and eliminate PRRSV, especially for candidate vaccine development that will potently induce both innate and adaptive immune response.

## Author Contributions

Y-DT and CS conceptualized the idea and generated the figures. All authors wrote the manuscript, contributed to the article, and approved the submitted version.

## Conflict of Interest

The authors declare that the research was conducted in the absence of any commercial or financial relationships that could be construed as a potential conflict of interest.
